# Genetically Predicted Neutrophil-to-lymphocyte Ratio and Coronary Artery Disease: Evidence from Mendelian Randomization

**DOI:** 10.1161/CIRCGEN.121.003553

**Published:** 2022-02-01

**Authors:** Arjen J. Cupido, Jordan M. Kraaijenhof, Stephen Burgess, F.W. Asselbergs, G. Kees Hovingh, Dipender Gill

**Affiliations:** 1 Department of Vascular Medicine, Academic Medical Center, Amsterdam University Medical Centers, University of Amsterdam, Amsterdam, The Netherlands; 2 Department of Cardiology, Division Heart & Lungs, University Medical Center Utrecht, Utrecht University, Utrecht, the Netherlands; 3 Division of Cardiology, Department of Medicine, University of California, Los Angeles, Los Angeles, CA; 4 Medical Research Council Biostatistics Unit, Cambridge Institute of Public Health, University of Cambridge, Cambridge, UK; 5 Department of Public Health and Primary Care, University of Cambridge, Cambridge, UK; 6 Institute of Cardiovascular Science, Faculty of Population Health Sciences, University College London, London, UK; 7 Health Data Research UK and Institute of Health Informatics, University College London, London, UK; 8 Novo Nordisk Research Centre Oxford, Old Road Campus, Oxford, UK; 9 Department of Epidemiology and Biostatistics, School of Public Health, Imperial College London, London, UK; 10 Clinical Pharmacology and Therapeutics Section, Institute for Infection and Immunity, St George’s, University of London, London, UK

**Keywords:** cardiovascular disease, inflammation, causality, NLR

Inflammation contributes to atherosclerosis and coronary artery disease (CAD). In order to help identify therapeutic targets, it is important to ascertain whether biomarkers associated with CAD risk are causal. In a recent meta-analysis of clinical trials, neutrophil-to-lymphocyte ratio (NLR) was associated with increased cardiovascular risk^1^. We investigate a potential causal nature of this relationship by performing Mendelian randomization (MR) analyses.

All participants provided prior consent for each study included, and the studies were approved by the relevant review committees. Non-public estimates are available from the corresponding author upon reasonable request. To identify genetic variants associated with NLR, we obtained published genome-wide association study (GWAS) summary data for neutrophil count and lymphocyte count in 361,194 European ancestry participants from http://www.nealelab.is/uk-biobank/, which were selected based on self-reported ancestry and genetic principal components. The propagation of error method was used to estimate the association of all available single-nucleotide polymorphisms (SNPs) with lymphocyte count subtracted from neutrophil count, resulting either in a positive value (which represents an increasing NLR) or a negative value (representing a decreasing NLR). Instruments were selected for Mendelian randomization by clumping all common SNPs (MAF > 0.01) at genome-wide significance (p<5×10^-8^) to pairwise linkage disequilibrium threshold r^2^ <0.01 (using European participants from the 1000Genomes project as reference). We estimated the association of each candidate instrument with NLR in individual participant data on 396,020 UK Biobank participants of white British descent with similar genetic ancestry (as reported by the UK Biobank resource), for whom neutrophil count, lymphocyte count and data on all selected SNPs were available and had no extreme NLR value (defined as either NLR = 0 or NLR = infinity)^2^. To estimate the association of each SNP with NLR, we used log-linear regression with adjustment for age, sex, the first 10 principal components of genetic ancestry, and measurement batch. We chose log-linear regression because NLR can only be positive, with some values close to zero, rendering normal linear regression inappropriate. Figure 1A shows the distribution of NLR, which is similar to the distributions from various clinical trials including CANTOS^1^.

Statistical power calculations for the minimum detectable odds ratio were performed for a power of 80% and type 1 error rate of 0.05^3^. We performed two-sample MR analyses, where we considered the following outcomes: CAD, myocardial infarction (MI), circulating C-reactive protein (CRP), interleukin 6 (IL-6) and fibrinogen levels (Figure). CRP, IL-6 and fibrinogen were considered to investigate the effect of NLR on inflammatory biomarkers^4^. Finally, to investigate the validity of the NLR instruments, we performed analyses with neutrophil count and lymphocyte count as outcomes. If data on a SNP was unavailable in one specific GWAS, we searched for an available proxy in high LD (r^2^ > 0.9) and if unavailable we omitted the SNPs for that specific analysis. All analyses were performed using the package TwoSampleMR in R v4.0.3. Results are presented per 1 unit increase in genetically-predicted log-transformed NLR.

Power calculations showed that we had 80% power to detect a minimum odds ratio of 1.07 for CAD. For MI, power calculations showed 80% power to detect an odds ratio of 1.08. After clumping and omitting multi-allelic SNPs, a total of 182 uncorrelated SNPs were selected as potential instruments in MR analyses given their genome-wide significant association with lymphocyte count subtracted from neutrophil count. In primary analyses (Figure 1B), we observed strong evidence of an association between genetically-predicted NLR and neutrophil count (2.66 % of white blood cell count (WBC), 95% CI 2.42, 2.90) and lymphocyte count (-0.59 % of WBC, 95% CI -0.83, -0.35). We did not observe evidence supporting a causal effect of NLR on CAD (0.91, 95% CI 0.69, 1.19), MI (0.91, 95% CI 0.67, 1.22), CRP (0.02 natural-log(mg/l) units, 95% CI -0.34, 0.39), IL-6 (-0.16 SD units, 95% CI - 0.49;0.17) or fibrinogen (0.07 log(g/L), 95% CI -0.18;0.32). In sensitivity analyses, we observed similar results using the MR Egger method. Excluding outliers, MR-PRESSO showed similar results to the primary inverse-variance weighted results, with the exception of CRP (Figure 1B).

This MR study did not identify evidence to support that NLR is causally related to risk of CAD and MI. Moreover, we did not find consistent evidence of a causal association of NLR on CRP, IL6 or fibrinogen levels. This contrasts with previous reports that have suggested a potential causal role for NLR in CAD, given the finding that both neutrophils and lymphocytes are involved in atherogenesi^1,5^. The discrepancy may be attributable to the associations identified in epidemiological studies arising due to confounding and reverse causation. However, even if not causally related to CAD, NLR could still be used as a predictive measure for cardiovascular disease risk. To what extent NLR has added value in future cardiovascular risk prediction over and above CRP levels remains to be fully explored.

Most data are retrievable from the public domain. Summary data for the genetic instrument are available from the corresponding author.

## Figures and Tables

**Figure 1:**
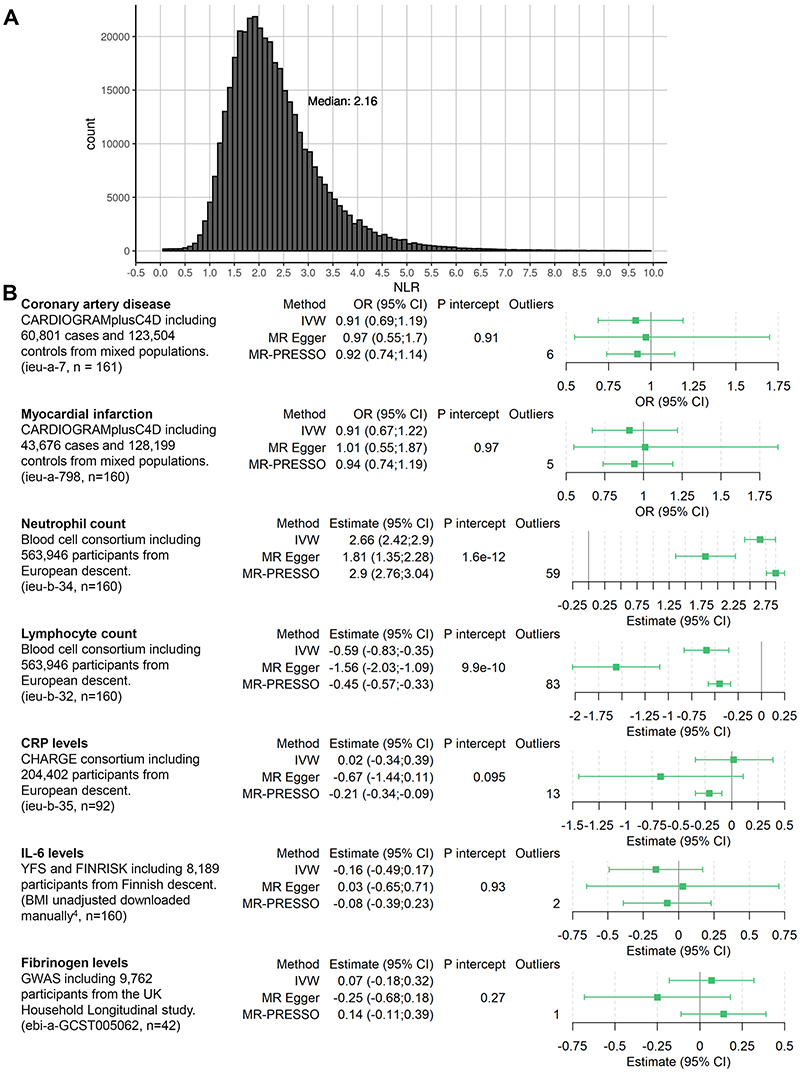
NLR distribution and forest plot A: Distribution of NLR in the UK Biobank. 767 values above 10 are not displayed for clarity purposes. B: Overview of included data and estimates for the Mendelian randomization association scaled to a 1 unit increase in genetically-predicted log-transformed NLR. Estimates for CAD and MI are depicted as odds ratio; neutrophil and lymphocyte counts as percentage of total white blood cell count; CRP as natural-log(mg/L); IL-6 as SD unit; and fibrinogen as log(g/L). n = number of SNPs of the instrument available in the outcome dataset and thus used for analysis. P intercept = Egger intercept p-value, Outliers = outliers removed by MR PRESSO. IVW = Inverse Variance Weighted method. ieu-X-XXX = MRC integrative Epidemiology Unit open GWAS project dataset code for use in TwoSampleMR package (https://gwas.mrcieu.ac.uk/).

## Non-standard Abbreviations and Acronyms

CAD: Coronary artery disease
CRP: C-reactive protein
GWAS: Genome-wide association study
IL-6: Interleukin-6
MAF: Minor allele frequency
MI: Myocardial infarction
MR: Mendelian Randomization
NLR: Neutrophil – to – Lymphocyte ratio
SNP: Single-nucleotide polymorphisms
